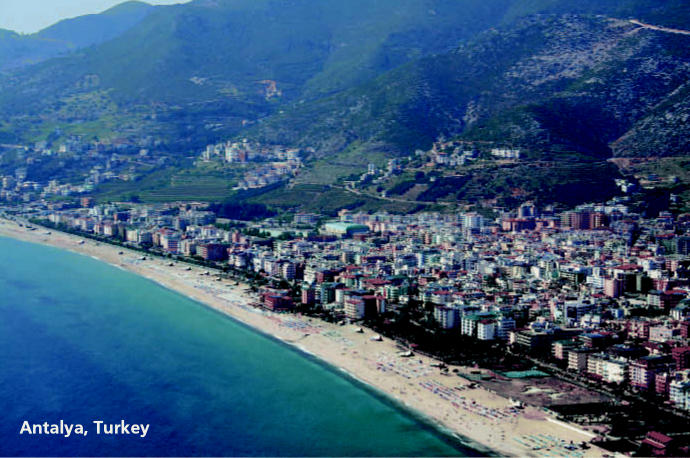# Change of Venue: Taking Environmental Mutagen Research to the Developing World

**DOI:** 10.1289/ehp.114-a696

**Published:** 2006-12

**Authors:** Tanya Tillett

The health of a nation could be said to depend upon the public health expertise of its scientists. In the United States and other developed areas of the world, it can be fairly simple to gain access to a variety of useful public health resources. But populations living in less-developed areas of the world often lack such knowledge and public health access. In 1987, two young scientists, William Au of the University of Texas Medical Branch (UTMB) and Wagida Anwar of Ain Shams University in Cairo, Egypt, decided to seek ways to raise the level of access to information and technical expertise regarding environmental health disparities in less-developed regions.

With a main goal of encouraging collaborations for growth of scientific knowledge and expertise as well as clinical applications in underserved areas, they focused on organizing an international conference highlighting the latest research and information on environmental mutagens. The fruit of that labor, the International Conference on Environmental Mutagens in Human Populations (ICEMHP) series, has become a boon to the scientific community in developing nations. The next conference, sponsored in part by the NIEHS, is scheduled for May 2007 in Antalya, Turkey.

NIEHS scientist Mike Waters, chairman of the ICEMHP International Advisory Board, says the institute has been a key participant in the conference series since its inception, noting that institute scientists have helped with organizing activities, and that NIEHS leaders have contributed personal and financial support. He also points out that the proceedings of the first two conferences were published in the October 1993 and May 1996 issues of *EHP Supplements*.

According to Waters, the mission of the NIEHS lends itself to sponsorship of the ICEMHP series. “[The NIEHS] fosters national as well as international efforts to reduce the burden of environmentally associated disease,” he explains. “This series of conferences certainly has the same objective. It represents an effective means of communicating and extending the NIEHS mission and its research findings to the developing world.”

Other past and current sponsors for the conference series include the U.S. CDC, Novartis Pharmaceutical Company, the U.S. Environmental Mutagen Society, the European Environmental Mutagen Society, the International Association of Environmental Mutagen Societies, the Turkish Society of Toxicology, Research Corporation, and the UTMB Department of Preventive Medicine and Community Health and Sealy Center for Environmental Health and Medicine.

## Accessible Science

In recalling the impetus for initiating the first conference, Au reflects, “If you like your work, you think about what else you can do to make a difference, so we were looking for what we could do to have an impact. We recognized that major conferences were held in developed countries, and we thought: why not replicate this in other regions with a need for environmental health outreach?”

With encouragement and support from their mentor, the late Frits Sobels of the University of Leiden, the Netherlands, the two young scientists saw the successful staging of the first ICEMHP in Anwar’s home-town of Cairo in January 1992. The Cairo conference was a noted success, with more than 200 attendees participating in symposia, oral presentations, poster sessions, and workshops. Following this conference, the Pan African Environmental Mutagen Society was formed, and Anwar was later named Egypt’s assistant minister of health.

The accomplishments of this first meeting paved the way for the development of more ICEMHP conferences. Subsequent meetings have been conducted in Prague, Czech Republic (1995), Bangkok and Khao Yai, Thailand (1998), and Florianopolis, Brazil (2003), with each stimulating public health programs and collaborations in the host regions. Au, who has remained the driving force behind the conferences, collaborates with scientists native to each region when organizing the proceedings, and gives priority to locations where the field of environmental mutagenesis is still in the developmental stages. He says one of the main goals in choosing each location is to make the opportunity for the exchange of scientific information available to more people, particularly those such as students in developing areas of the world, who might not have the means to fly to cities across the globe where major conferences are usually held.

Students, researchers, practicing clinicians, and each host country’s Ministry of Health are among the regular invitees to each ICEMHP. Students in particular are actively recruited; they are offered reduced registration fees, and are provided with copies of ICEMHP monographs they might not otherwise be able to afford––monographs containing valuable environmental health information they can consult as they develop strategies for positive impacts on health in their home regions.

Radim Šrám, director of the Laboratory of Genetic Ecotoxicology at the Institute of Experimental Medicine in Prague, Czech Republic and co-organizer of the 1995 ICEMHP, sees the meetings as ideal platforms for the exchange of environmental health information to effect change in each participant’s home region. “Organizers try to bring to these meetings young scientists from developing countries, to stimulate their interest in the topics to be used in their countries. . . . These conferences become a friendly forum to understand the level of science in different regions as well as the differences among the exposure to carcinogens in different countries.” This information, he says, “may be later used for regulatory changes.” He also notes that conference activities always highlight the latest environmental mutagen research, including information on new methods and biomarkers for human studies, and how they should be processed and interpreted.

## A Rewarding Challenge

Although organizers encounter a variety of issues unique to less-developed areas of the world during the planning years between each conference, they are usually able to work around these challenges. The conferences have had a steady increase in the rate of attendance over time, even though they have taken place during politically, socially, and economically challenging times. For example, the 2003 conference in Brazil had a record attendance of more than 600 individuals despite occurring during a time of turmoil in the Latin American financial market and at the height of worldwide concern about sudden acute respiratory syndrome and the risks of international travel.

The conference budget also presents its own challenge. Unlike most established conferences, which are sponsored by professional organizations and societies, each ICEMHP depends wholly upon its organizers in seeking financial support. Invited speakers help defray some of the costs by paying their own travel expenses. Organizers have also received funding from outside sponsors such as individual environmental health experts and entities who see the intrinsic value in supporting the meetings.

With four successful conferences behind him and the May 2007 conference coming up soon, Au is looking ahead to the continued fostering of sustainable programs and initiatives on the local level in developing countries. Locating the conferences in less-developed regions remains the cornerstone: ensuring that young scientists in developing regions have access to current thinking not only helps them improve their own knowledge and technical skills, but also fosters a new generation of environmental health policy makers, says Au.

Furthermore, each meeting to date has helped forge collaborative efforts that have stimulated the development of local environmental mutagen societies and projects dedicated to improving local environmental health over the long term. Au says that connecting with environmental health decision makers in underserved areas to determine and address important regional issues will continue to help shape the ICEMHP series.

## Figures and Tables

**Figure:**